# Effects of Maternal Hypoxia during Pregnancy on Bone Development in Offspring: A Guinea Pig Model

**DOI:** 10.1155/2014/916918

**Published:** 2014-05-14

**Authors:** Alice M. C. Lee, Janna L. Morrison, Kimberley J. Botting, Tetyana Shandala, Cory J. Xian

**Affiliations:** ^1^Sansom Institute for Health Research, School of Pharmacy and Medical Sciences, University of South Australia, City East Campus, GPO Box 2471, Adelaide, SA 5001, Australia; ^2^Discipline of Physiology, School of Medical Sciences, University of Adelaide, Adelaide, SA 5005, Australia

## Abstract

Low birth weight is associated with reduced bone mass and density in adult life. However, effects of maternal hypoxia (MH) on offspring bone development are not known. *Objective*. The current study investigated the effects of fetal growth restriction induced by MH during the last half of gestation on bone structure and volume in the offspring of the fetus near term and the pup in adolescence. *Methods*. During 35–62-day gestation (term, 69d), guinea pigs were housed in room air (21% O_2_; control) or 12% O_2_ (MH). Offspring femur and tibia were collected at 62d gestation and 120d after birth. *Results*. MH decreased fetal birth weight but did not affect osteogenic potential pools in the fetal bone marrow. Histological analysis showed no effects of MH on tibial growth plate thickness in either fetal or postnatal offspring, although there was increased VEGF mRNA expression in the growth plate of postnatal offspring. MH did not change primary spongiosa height but lowered collagen-1 mRNA expression in postnatal offspring. There was increased mRNA expression of adipogenesis-related gene (FABP4) in bone from the MH postnatal offspring. *Conclusion*. MH during late gestation did not change the pool of osteogenic cells before birth or growth plate heights before and after birth. However, MH reduced expression of bone formation marker (collagen-1) and increased expression of fat formation marker (FABP4) in postnatal offspring bone.

## 1. Introduction

Body composition and musculoskeletal development begin in embryonic life, when bone and muscle develop from the mesodermal layer. Longitudinal bone growth before and after birth takes place in the growth plate located at the epiphyses of long bones via an endochondral ossification process in which a hypertrophic cartilage template is made, calcified, and invaded by blood vessels and then converted to trabecular bone through the action of bone forming cells, osteoblasts, and bone resorbing cells, osteoclasts [1]. Bone mass and bone size increase throughout childhood, reaching their peak at the end of adolescence, when growth plates are closed [2, 3]. While optimal bone growth achieved during childhood is critical both for ensuring optimal development and protection from fractures during childhood, evidence is growing that peak bone mass is also an important contributor to bone mass and strength in later life [4]. Peak bone mass is a predictor of the age of onset of osteoporosis and a determinant of osteoporotic fracture risk in the elderly [5–8], a skeletal disorder associated with a progressive decrease in bone mineral density (BMD) and an increase in bone fragility and susceptibility to fractures.

There are a range of endocrine and paracrine factors that regulate bone growth [1]. Alterations to the diet of pregnant animals can produce lasting changes in the phenotype, physiology, and metabolism of the offspring [9–12]. This results in a range of adaptations that occur in response to the reduced substrate supply and have been shown to permanently program its physiology and metabolism [11, 13]. Fetal growth restriction can be caused by maternal undernutrition, maternal hypoxia (MH), maternal smoking, and placental insufficiency [13, 14]. Placental insufficiency and MH both result in fetal hypoxemia during the latter part of pregnancy [15, 16]. Studies have shown that birth weight is associated with bone mass, and low birth weight is related to a low bone mineral content in adults and increased fracture risks later in life [17–19]. However, the role of hypoxia on bone formation/remodelling is unclear.

In multicellular species, local oxygen partial pressure represents the functional regulator of oxygen homeostasis in the lung, blood, and other tissues. Insufficiency of oxygen can result in a failure to generate sufficient ATP to maintain essential cellular functions [18]. In physiological circumstance or pathological conditions, a program of gene expression changes is induced by hypoxia-inducible factor-1 (HIF-1). HIF-1 controls hypoxic expression of genes with metabolic functions such as glucose transport and metabolism and angiogenic factors like vascular endothelial growth factor (VEGF). As a subunit of HIF, HIF-*α* is a hypoxically responsive component. Studies have suggested that induced hypoxia in mouse and rabbit embryos leads to a variety of developmental defects that occur in places including the heart and vertebral column [17, 19, 20]. Furthermore, smoking during pregnancy, which causes fetal hypoxemia, may suppress bone matrix synthesis and mineralisation during gestation with lower osteocalcin levels in the umbilical blood in neonates of smoking mothers [20].* In vitro* studies with human mesenchymal stem cells exposed to reduced oxygen tension demonstrated delayed osteoblast maturation, shown by reduced mRNA expression of osteocalcin and type 1 collagen (Cola1). However, it appears that the timing and the extent of the hypoxia influence the type of developmental process that is disrupted [21].

We hypothesised that MH would result in decreased bone development in the fetus and thus reduced bone mass in adolescence. The current study examined the effects of fetal growth restriction induced by MH during the last half of gestation on bone structure and volume in the offspring before and after birth.

## 2. Materials and Methods

### 2.1. Animals

All procedures were approved by the Animal Ethics Committees at the University of South Australia (IMVS) and the University of Adelaide. IMVS tricoloured guinea pigs were individually housed at 18–22°C and in a 12/12 light cycle. All animals were fed standard laboratory rabbit/guinea pig chow (Laucke Mills, Daveyston, Australia) with* ad libitum* access to water supplemented with 0.5 g/L Vitamin C. Breeding females (sows) were weighed and given a known weight of food three times weekly. The remaining food was weighed to determine food intake per gram of body weight. After 4–6 weeks of controlled feeding, breeding females were placed with a male for 24 h and were mated. Females in oestrus were placed with a male overnight and pregnancy was detected by the presence of a vaginal copulatory plug the following morning and a failure to return to oestrus in the subsequent cycle [22, 23].

### 2.2. Experimental Protocol and Specimens

At 35d gestation, pregnant sows were randomly assigned to control or MH groups. Sows assigned to MH were housed in 12% oxygen (half of normal % of oxygen in air [24]) with* ad libitum* access to food. For fetal studies (*n* = 5), MH continued until sows and fetuses were humanly killed at 62d gestation (term, ~69d) with an overdose of sodium pentobarbitone (325 mg/mL pentobarbitone sodium; Virbac Pty Ltd., Peakhurst, Australia). For postnatal studies (*n* = 5), MH continued until 65d gestation, whereupon pregnant sows were returned to normoxia (21% oxygen in air), to ensure pups were born into normoxia. All sows and pups were exposed to normoxia with* ad libitum* access to food during lactation and postweaning. Pups were weaned at 28d and subsequently housed in same sex pairs until humanely killed at 120d (adulthood [25]) with a sodium pentobarbitone overdose as above.

The left tibias from each fetus and pup were collected, fixed in 10% formalin, and decalcified in Immunocal solution (Decal Corporation, Tallman, NY) for 14d at 4°C prior to being bisected longitudinally and processing for paraffin wax embedding and sectioning (4 *μ*m). The right tibia was used to collect growth plate and metaphysis bone samples that were frozen in liquid nitrogen and stored at −80°C until being used for RNA extraction and gene expression analysis. Bone marrow cells were collected from the fetal femurs by flushing with basal cell culture medium as described [26].

### 2.3. Histomorphometric Analysis of Growth Plate and Metaphyseal Bone

To examine treatment effects on bone growth, bone structure, and volume, H&E-stained proximal tibial sections were used for basic histomorphometric measurements including growth plate thickness, primary spongiosa height, and secondary trabecular bone volume (BV/TV) as previously described [27, 28] and total adipocyte counts (number/mm^2^ bone marrow area) in lower secondary spongiosa as previously described [26].

### 2.4. Colony Forming Unit-Fibroblast Assay

To determine treatment effects on the size of the osteoprogenitor cell pool, a colony forming unit-fibroblast (CFU-f) assay and alkaline phosphatase (ALP, an osteoprogenitor cell marker) staining were performed with bone marrow cells obtained from fetal femurs as described [29]. Briefly, isolated bone marrow cells were plated at 2 × 10^6^ mononuclear cells (MNCs)/well in 6-well tissue culture dishes with complete medium (*α*-MEM supplemented with 10% FBS, 50 *μ*g/mL Pen/Strep, 15 mM HEPES, and 100 *μ*M L-ascorbic acid-2-phosphate). Cells were cultured for 14d with media changed every 3d. At the end of culture, cells were fixed with 4% paraformaldehyde and stained for ALP using a kit (Roche Biochemicals, Sydney, Australia). The number of positive colonies (osteoprogenitor cell pool in the bone marrow) was expressed as CFU-f ALP^+^ colonies per 2 × 10^6^ MNCs.

### 2.5. Real-Time Reverse Transcriptase Polymerase Chain Reaction (RT-PCR)

Frozen growth plate and metaphyseal bone specimens were ground to fine powder with liquid nitrogen using a mortar and pestle, and total RNA was extracted using TRI reagent (Sigma, NSW, Australia). To generate the template for PCR amplification, 2 *μ*g of metaphyseal RNA was reverse transcribed into cDNA using the high capacity RNA-to-cDNA kit (Applied Biosystems, Foster City, CA). All PCR primers (Table 2) were designed using guinea pig DNA sequences and NCBI PRIMER BLAST software and supplied by GeneWorks (Adelaide, SA, Australia). For quantitative RT-PCR of genes of interest (Table 2), a real-time PCR reaction of 10 *μ*L was set up using SYBR1 Green master mix (Applied Biosystems). For each cDNA sample, quantitative PCR assays were run on a 7500 Fast Real-Time PCR System (Applied Biosystems) in duplicate. From the amplification curves, relative expression was calculated using the comparative 2^−ΔΔCT^ method, with cyclophilin A serving as the endogenous control as described [26].

### 2.6. Statistical Analysis

Data are presented as means ± SEM and were analysed using an unpaired Students'* t*-test and were nested for litter using STATA10 (StataCorp, TX, USA). *P* < 0.05 was considered significant.

## 3. Results

### 3.1. MH Decreased Offspring Body Weight

MH resulted in reduced fetal body weight at 62d gestation compared to fetuses from control mothers (*P* < 0.05; Table 1). However, by 120d after birth, there were no differences in body weight between treatment groups.

### 3.2. MH Did Not Change Growth Plate Thickness

**Figure 1:**
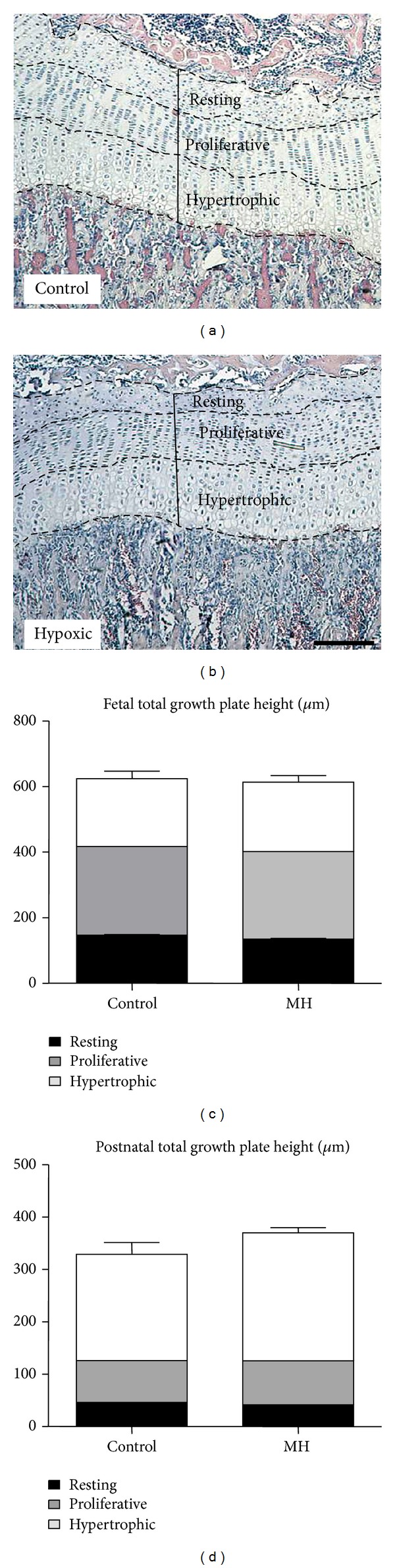
Effects of MH on total thickness and zonal heights in the growth plate of offspring. H&E-stained tibial sections (showing growth plate height) of 120d postnatal guinea pigs from a control (a) and a hypoxic (b) mother, respectively. The dashed lines in (a) and (b) arbitrarily separate the three individual growth plate zones. (c) Total growth plate thickness and zonal heights (resting, black bar; proliferative, grey bar; hypertrophic, white bar) of 62d gestation fetal guinea pigs from control and hypoxia-treated mothers. (d) Total growth plate thickness and zonal heights of 120d postnatal guinea pigs from control and hypoxia-treated mothers (*n* = 5). Scale bar in (a) = 250 *μ*m and applies to (b).

Histomorphometric measurements demonstrated that MH did not alter tibial growth plate total thickness in either fetal or postnatal offspring compared to controls (Figures 1(a)–1(d), *P* > 0.05). There was no effect of MH on zonal heights of the growth plate in either the fetal or postnatal tibial bone (Figures 1(c) and 1(d)).

### 3.3. Gene Expression of Chondrocyte Maturation Markers

**Figure 2:**
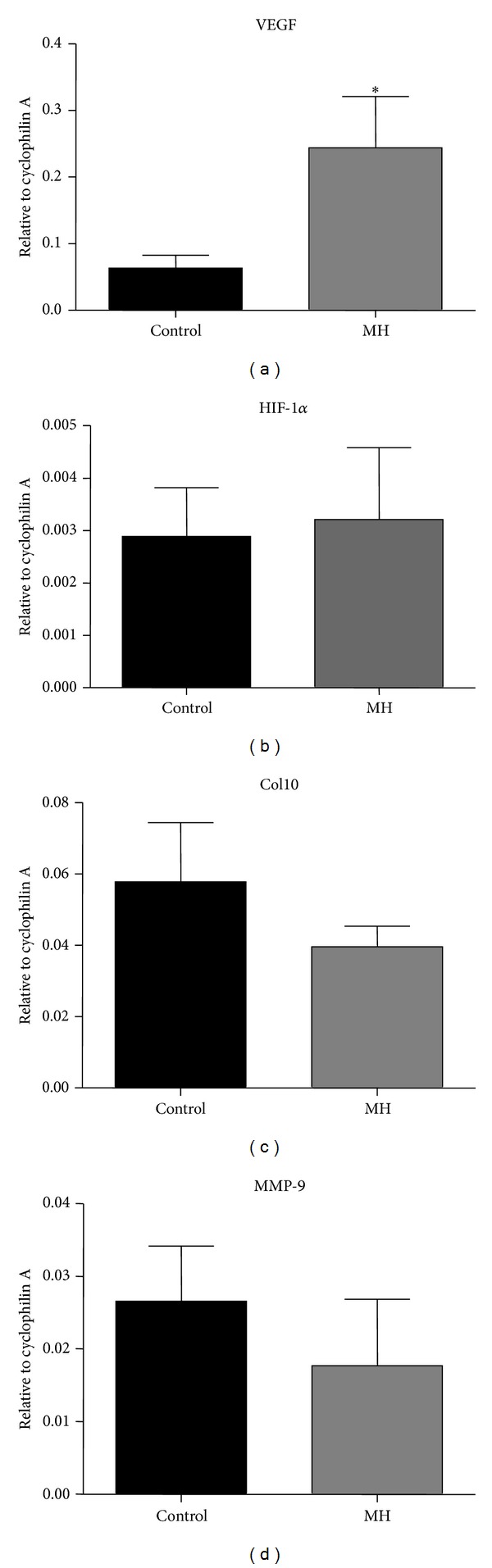
Effects of MH on mRNA expression of genes in growth plate in the postnatal offspring. Quantitative real-time RT-PCR expression data for VEGF (a), HIF-1*α* (b), Col10 (c), and MMP-9 (d) are expressed as relative to internal standard cyclophilin A.  *P < 0.05 when compared to control.

The mRNA expression of the key angiogenic growth factor, VEGF, was significantly higher in the growth plate of postnatal offspring exposed to MH compared to controls (Figure 2(a)). However, there were no differences in the mRNA level of hypoxia-induced factor (HIF-1*α*) between offspring from the MH and control groups (Figure 2(b)). MH did not affect levels of mRNA expression of collagen type X (Col10) and matrix metalloprotease-9 (MMP-9), both involved in the maturation of the growth plate cartilage and its bone conversion (Figures 2(c) and 2(d)).

### 3.4. MH Did Not Change Primary Spongiosa Height of Metaphysis

**Figure 3:**
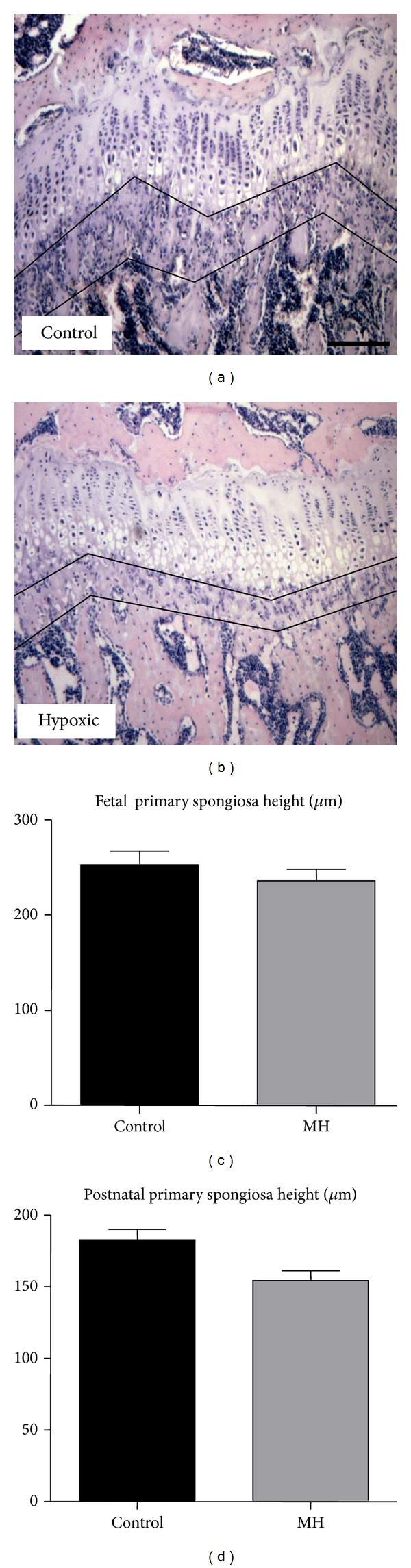
Effects of maternal hypoxia on offspring proximal tibia primary spongiosa height. (a) and (b) H&E-stained tibial sections (primary spongiosa shown between two tracing lines) of 120d postnatal guinea pigs from a control and a hypoxic mother, respectively. (c) Primary spongiosa height of (c) 62d gestation fetal and (d) postnatal 120d guinea pigs from control and hypoxia-treated mothers (*n* = 5). Scale bar in (a) = 1 mm and applies to (b).

To examine the effect of MH on endochondral bone formation, heights of primary spongiosa (newly derived from calcified growth plate cartilage) were measured (Figures 3(a) and 3(b)). MH did not affect the primary spongiosa height of fetal (Figure 3(c)) or postnatal offspring compared to controls (Figure 3(d); *P* > 0.05).

### 3.5. Effects of MH on Bone and Bone Marrow Fat Volumes

**Figure 4:**
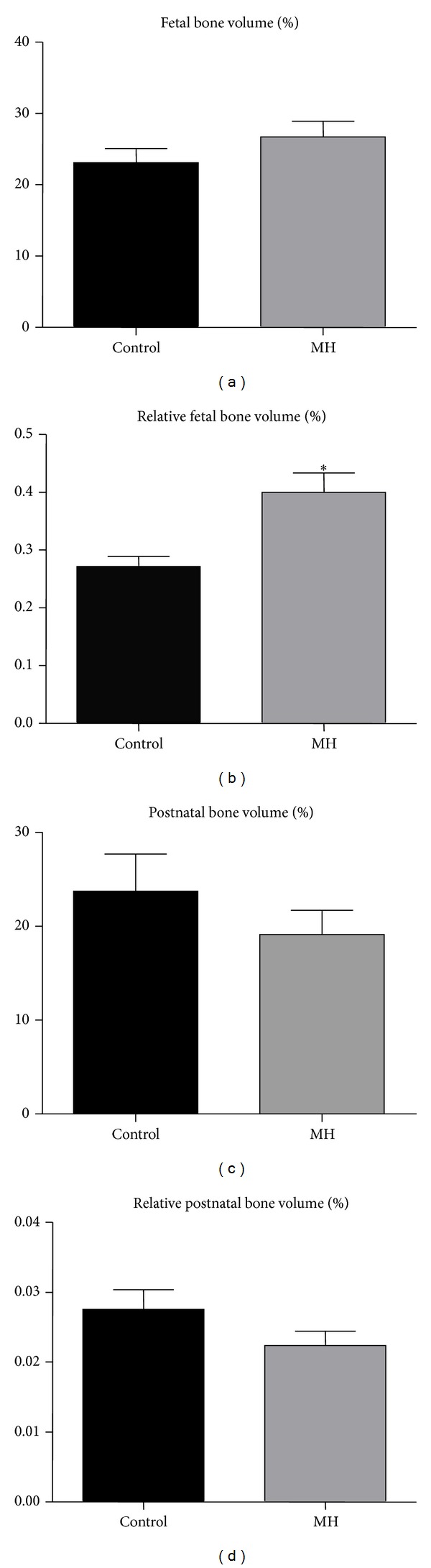
Effects of MH on tibial trabecular bone volume as measured by histomorphometry (BV/TV, %). (a) BV/TV of fetal bones from control and hypoxia-treated mothers. (b) Relative BV/TV (adjusted for total body weight) in fetal guinea pigs (%/g). (c) BV/TV of bones of 120d postnatal guinea pigs from control and hypoxia-treated mothers. (d) Relative BV/TV in postnatal guinea pigs (adjusted for total body weight, %/g). (*n* = 5,  *P < 0.05 when compared to control).

Metaphyseal trabecular bone volume was analysed in the secondary spongiosa, which is composed of enlarged mineralized bony trabeculae modelled or remodelled from the primary spongiosa. There were no significant differences in bone volume (BV/TV %) in fetal (Figure 4(a)) or postnatal (Figure 4(c)) offspring of MH compared to control mothers. When the bone volume was adjusted for body weight (bone volume/body weight), the relative bone volume was higher in MH compared to control fetuses (Figure 4(b); *P* < 0.05), but not postnatal offspring (Figure 4(d)).

**Figure 5:**
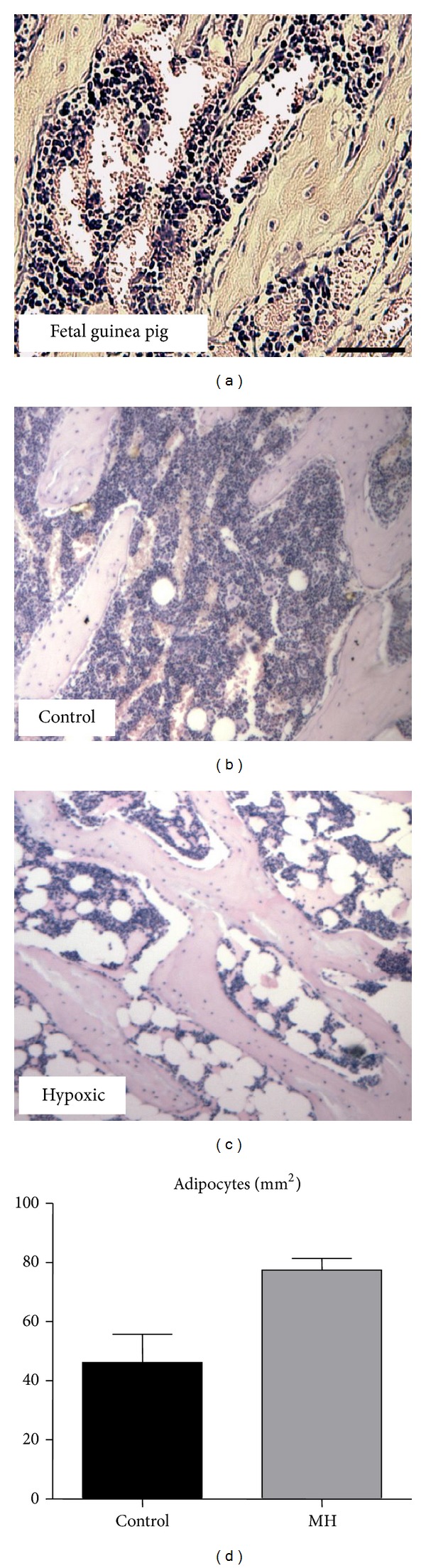
Effects of MH on numbers of adipocytes in the lower secondary spongiosa of tibial bone. (a) Bone marrow in fetal guinea pigs does not contain adipocytes (a section from a control fetal guinea pig). Bone marrow showing presence of adipocytes in a day 120 postnatal guinea pig from a control (b) and a hypoxic mother (c). (d) Bone marrow adipocytes/mm^2^marrow area from control and hypoxia-treated mothers. *n* = 5. Scale bar in (a) = 500 *μ*m and applies to (b) and (c).

There were no adipocytes in the bone marrow in fetal guinea pigs (Figure 5(a)). There was no effect of MH on marrow adiposity in postnatal offspring of MH compared to control mothers (Figures 5(b), 5(c), and 5(d)).

### 3.6. MH Did Not Change Osteogenesis but Increased Adipogenesis-Related Gene Expression

**Figure 6:**
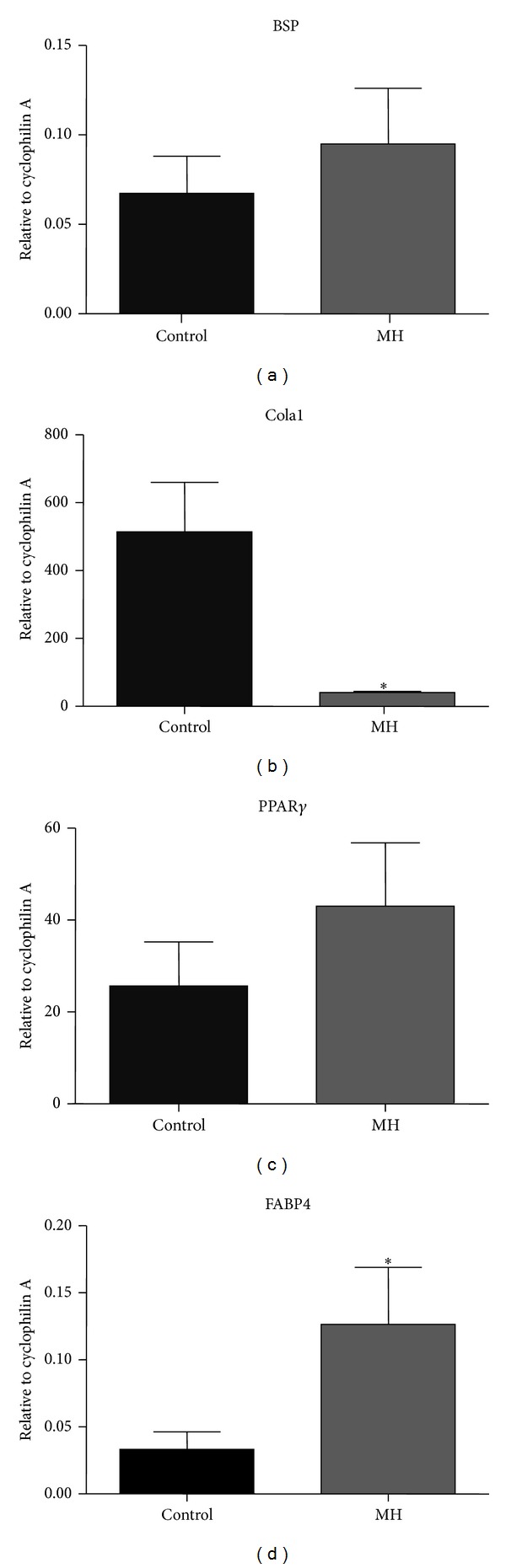
Effects of MH on mRNA expression of genes in the metaphysis bone of postnatal offspring. Quantitative real-time RT-PCR expression data for BSP (a), Cola1 (b), PPAR*γ* (c), and FABP4 ((d), *P* = 0.05) are expressed relative to internal standard cyclophilin A.  *P < 0.05 when compared to control (*n* = 5).

Bone sialoprotein (BSP) gene expression in the metaphysis of postnatal offspring was not affected by MH (Figure 6(a)). However, MH offspring had a lower level of collagen-1 (Cola1) mRNA expression compared to control offspring after birth (*P* < 0.05; Figure 6(b)). While there were no significant changes in mRNA expression of the adipogenic transcription factor, peroxisome proliferator-activated receptor gamma (PPAR*γ*; Figure 6(c)), MH induced an increase in mRNA expression of fatty acid binding protein 4 (FABP4) in the postnatal offspring (Figure 6(d)).

### 3.7. MH Did Not Change the Size of the Bone Marrow Osteoprogenitor Cell Pool

**Figure 7:**
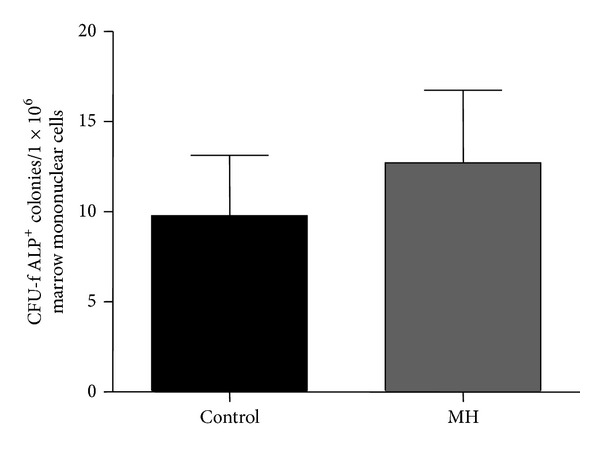
Effects of MH on the osteoprogenitor cell pool in bone marrow of fetal guinea pigs. Data showing the number of CFU-f colonies stained positive for alkaline phosphatase (ALP) in total bone marrow mononuclear cell population (values are means ± SEM, *n* = 5).

CFU-f assay and ALP staining were carried out with bone marrow samples from the fetal guinea pigs to examine the osteoprogenitor cell pool in the bone marrow (Figure 7). There were no significant differences in the osteoprogenitor pool size in fetal offspring of mothers from both groups.

## 4. Discussion

One of the main consequences of fetal growth restriction is low birth weights with low bone mass [30, 31]. Population studies have demonstrated that there are associations between weight at 1 year and adult bone mineral contents at lumbar spine and femoral neck as well as adult bone mass [19, 32–34]. There is also evidence that fetal growth restriction results in an increased risk of osteoporotic fracture [18, 35], suggesting that the lack of nutrients early in life may compromise adult skeleton. The present study has shown that MH caused a significant reduction in fetal weight. MH did not change the growth plate thickness, despite increased mRNA expression of VEGF in postnatal offspring. Histological and gene expression studies suggest that MH also reduced bone formation potential and a potential increase in bone marrow adiposity (increased FABP4 mRNA expression) in postnatal offspring in this model.

### 4.1. Effects of Maternal Hypoxia on Histology and Gene Expression in the Growth Plate of Postnatal Offspring

MH caused fetal growth restriction, but offspring weight was accelerated in postnatal life such that by 120d of age offspring in the control and MH group were the same weight. However, MH had no effect on tibial growth plate thickness or primary spongiosa heights, two regions that are mainly responsible for bone lengthening and bone mass accumulation before or after birth.

There was no change in HIF-1*α* mRNA expression in MH postnatal offspring. There were, however, changes in the expression of some but not all genes that are regulated by HIF-1*α*. Interestingly, there was a significant elevation in the expression of VEGF in MH postnatal offspring. VEGF is induced by the hypoxia-mediated control of gene expression (particularly HIFs) [36, 37] and induces angiogenesis. Our finding of increased VEGF expression is consistent with a previous study where hypoxia increased VEGF production in osteoblasts [38, 39]. HIF-mediated functions assure the differentiation of progenitor cells when the cells are exposed to hypoxic conditions during chondrogenic or osteogenic differentiation in embryo development [40]. It has been shown that placental insufficiency can result in nonphysiological hypoxia, and it appears that timing and extent of the deficiency determine the type of developmental process that is disturbed [41]. In the current study, levels of expression of HIF-1*α* in the growth plate of the postnatal offspring of hypoxic mothers remained unchanged, suggesting that the extent of hypoxia did not cause an upregulation of the hypoxic responsive transcription factor. However, further work investigating the protein levels of HIF-1*α* is required to understand the mechanism behind the observed increase in VEGF mRNA expression. In addition, MH did not affect expression of gelatinase or MMP-9 (another downstream target gene of HIF-1*α*) in the growth plate of offspring. Both VEGF and MMP-9 have been shown to play a central role in embryonic bone development and bone remodelling. The lack of effects of MH on HIF-1*α* and MMP-9 expression may be related to the extent and duration of MH of the current study which may not have yielded an adequate* in utero* stimulus able to alter bone development in the offspring. In addition, the lack of effects could also be due to normalisation of their expression postnatally.

Consistent with the lack of an effect of MH on MMP-9 mRNA expression and the height of the growth plate and primary spongiosa, MH did not change the expression of another hypertrophic cartilage marker Col10 in the growth plate of fetal or postnatal offspring. Thus, while it is difficult to determine whether MH in the current study may have delayed chondrocyte hypertrophy and endochondral bone development, it is possible that MH of a greater severity or a longer duration may have had a significant effect. Although* in vitro* studies have demonstrated that high oxygen tensions favour the hypertrophic differentiation of chondrogenic cultures or tissues [42, 43] associated with a possible increase in the expression of the hypertrophic cartilage marker genes, MMP13, and collage type 10 [44], a possibility for the lack of significant differences in the current model is adaptation, which has been previously shown in rabbit heart [45].

### 4.2. Effects of Maternal Hypoxia on Bone Structure in the Postnatal Offspring

There was no effect of MH on trabecular bone histological measurements in the long bones of postnatal offspring, which may further support the importance of the postnatal environment in contributing to the growth and development of the offspring. Some of the negative effects of fetal growth restriction on postnatal health can be rescued by the provision of a normal lactation environment [46], demonstrating that the postnatal environment is also influential in long-term bone health outcome [46]. The variable long-term effects of low birth weight on bone mineral density or hip fracture risk later in life suggest other contributing factors such as physical activity and nutrition may have a more substantial role in determining hip fracture risk [47]. These studies further suggest that bone mass at adulthood rather than at adolescence may be more important in determining the effects of MH and the onset of osteoporosis.

### 4.3. Effects of Maternal Hypoxia in the Bone Marrow Adiposity

MH in the current model did not change adipocyte contents or expression of the key adipogenesis-related gene PPAR*γ*; there was an increase in the mRNA expression of FABP4 and decrease in the mRNA expression of the bone formation marker Cola1 (despite a lack of change in bone volume fraction) in the metaphysis bone. These results suggest that although the bone volume fraction may have recovered from the* in utero* hypoxia as they were placed in normal normoxic environment postnatally, MH may have caused some permanent damage in the metaphysis that may have consequences later in life. Adipocytes and osteoblasts share a common stromal precursor in the bone marrow [48] and it has been suggested that there is a potential switch in differentiation of bone marrow stromal progenitor cells towards adipogenesis at the expense of osteogenesis in response to damages/stress [49]. This suggests that this mechanism may be present in the MH offspring, which is supported by the increase in mRNA expression of the adipogenesis-related gene FABP4 and the reciprocal reduction in the expression of the osteogenesis gene Cola1 in the bone. Previously, long-term MH in sheep increased mRNA expression of PPAR*γ* and its coactivator (PGC1*α*) mRNA in perirenal adipose tissue in late gestation [50], suggesting that there is an effect of hypoxia on adipogenesis in both bone marrow and extraosseous adipose tissues. These studies suggest that MH in the current setting may not be as detrimental to the bone and bone marrow as demonstrated by the lack of effects of MH on fetal guinea pigs.

### 4.4. Effects of Maternal Hypoxia on the Size of Fetal Bone Marrow Osteoprogenitor Cell Pool

MH did not change bone histomorphometry in fetal offspring, suggesting that osteogenic differentiation potential of bone marrow progenitor cells (ALP^+^ CFU-F colonies formed) was not affected by MH. The effect of MH on the osteogenic potential in the fetal offspring has not previously been studied. Several* in vitro* studies have demonstrated a negative effect of hypoxia on cell growth [39, 51] and osteogenic differentiation [21, 51], while others have shown a positive outcome for osteoblastic differentiation [52]. Hypoxia (2% oxygen) inhibited proliferation and delayed osteoblast differentiation of immature calvarial osteoblast precursors from neonatal rats [53]. In addition, hypoxia inhibited mineralised nodule formation by osteoblasts at both early and late stages of culture, which recovered well when cells were transferred to normal O_2_ levels after early exposure [53]. However, Salim et al. found difference between 21% O_2_ and 2% O_2_ conditions on differentiation potential of osteoblasts but diminished osteoblastic differentiation at 0.02% O_2_ [21]. These inconsistent studies suggest that effects of exposure to hypoxia of mesenchymal stem cells or osteoblastic cells may be time and dose dependent.

In summary, some previous studies have suggested that adverse influences, arising in fetal life or immediately after birth, may have an important effect on later risk of lower bone mass or osteoporotic fracture. We have demonstrated that MH during pregnancy in this model does not change growth plate and bone structures in offspring before and after birth. However, gene expression studies suggest that MH may have damaged the bone as demonstrated by an upregulation of genes involved in fat formation and a reciprocal downregulation of the genes involved in bone formation despite the lack of obvious changes in bone volume and adipocyte content in the postnatal bone marrow in this model. Further studies investigating the effects of maternal insufficiencies during the different stages of prenatal development and postnatal growth in offspring would increase our understanding of the consequences on offspring bone development and identify the crucial time points whereby changes in growth plate, bone, and bone marrow can be observed. Further investigations should be performed to determine if intrauterine insults can be rescued with postnatal nutritional interventions which may lead to the prevention of adult diseases such as osteoporosis.

## 5. Limitations

The current study has raised many questions which require further investigations. As the current study was limited to only observe the effects of maternal hypoxia at one point after birth, a future study should be carried out using the current model but with different observation time points such as 1, 3, and 5 months postnatally. This will further explain the influence of maternal hypoxia on bone development at different stages of skeletal development postnatally. Furthermore, although the bone volume in the current model was found to be not compromised by maternal hypoxia, biomechanical studies looking at the breaking strength of long bones as well as measurements of bone mineral contents (as measured by the ash weight of bone samples) should be employed to fully understand the effects of maternal hypoxia on the bone development in their offspring. Moreover, further studies should be carried out to investigate the proliferation and differentiation potentials of the marrow progenitor cells isolated from the offspring of hypoxic mothers. In addition, the protein expression of HIF-1*α* should also be investigated as it provides a clearer understanding as to whether a hypoxic environment in the bone marrow environment would have been created by maternal hypoxia in bone. Similarly, the sources (or cell types) of expression of key factors particularly RANKL, TNF-*α* and IL-1, and OCN should be investigated (e.g., by immunohistochemistry) to get more insights into osteoclastogenic and osteogenic potentials of the offspring following maternal hypoxia.

## Figures and Tables

**Table 1:** Effects of MH on body weights of fetal guinea pigs at 62 d gestation and postnatal guinea pigs at day 120. Values are means (± SEM), *n* = 5.

Treatment	Fetal Guinea Pig		Post natal Guinea Pig	
	Control	Hypoxic	Control	Hypoxic
Body weight, grams (sem)	84.4 (3.5)	66.9* (2.2)	869.2 (50.9)	858.4 (44.3)

*P < 0.05 when compared to control.

**Table 2:** Primer pairs used in this study.

Gene	Forward primer	Reverse primer
Cyclophilin A	ATCTGCACTGCCAAGACTGA	TGGTGATCTTCTTGCTGGTC
MMP-9	AGCCCTGCGCGTTTCCCTTC	CCTGGCGACCCTCGGTGGTA
ColX	TCGGGCCGCCAGGTATTCCA	AGACCCGGCCTTTGGCCTG
HIF1-a	GGGCCGGCTCCCCTACTGTC	CACCGTGCAGGTCCCACCCC
PPAR*γ*-1	TAGAGCCTGCATCGCCCCCA	ACACACGCGGCACTCGATGG
FABP4	TGGCATGGCCAAACCCAGCC	TGCGGTGACTTCATCGAATTCCTGG
BSP	CACCACAGCAGGCGCTACCC	GTTCCCGGGTGGGAGGGTGT
Cola1	CCGGTCCTGCTGGTCCTGCT	GCCTTGTCACCACGGGGACCT

## Abbreviations

MH: Maternal hypoxia
BMD: Bone mineral density
HIF-1: Hypoxia-inducible factor-1
VEGF: Vascular endothelial growth factor
Cola1: Type 1 collagen
BV/TV: Trabecular bone volume
CFU-f: Colony forming unit fibroblast
MNCs: Mononuclear cells
RT-PCR: Real-time reverse transcriptase polymerase chain reaction
Col10: Collagen type X
MMP-9: Matrix metalloprotease 9
PPAR*γ*: Peroxisome proliferator-activated receptor gamma
FABP4: Fatty acid binding protein 4.
